# A Process Parameter Design Method for Improving the Filament Diameter Accuracy of Extrusion 3D Printing

**DOI:** 10.3390/ma15072454

**Published:** 2022-03-26

**Authors:** Kaicheng Yu, Qiang Gao, Lihua Lu, Peng Zhang

**Affiliations:** 1 School of Mechatronics Engineering, Harbin Institute of Technology, Harbin 150001, China; ykclwzy@163.com (K.Y.); zp@hit.edu.cn (P.Z.); 2 Research Center of Precision Equipment and Technology, Chongqing Research Institute of HIT, Chongqing 400000, China; 3 School of Astronautics, Harbin Institute of Technology, Harbin 150001, China

**Keywords:** extrusion 3D printing, printing accuracy, filament diameter, process parameter, rheological property

## Abstract

Process parameters have a significant impact on the filament diameter of extrusion 3D printing. To precisely control filament diameter, this paper proposes a novel method based on experiments to guide process parameter design. Additionally, an extrusion 3D printing device was developed, by which the influence of crucial process parameters and rheological properties on the diameter of printed filaments could be investigated experimentally and theoretically. Furthermore, poly (l-lactide-co-ε-caprolactone) (PLCL) was used as a case study to detail the design procedure of the proposed method. The printable range of the process parameters for PLCL was acquired, and a fitting surface for the experimental data was calculated to guide the process parameter design. According to the results of the experiment, by adjusting the process parameters, PLCL filaments with five different diameters of 120, 130, 140, 150, and 160 μm can be fabricated with a 100 μm nozzle. The deviations between the actual filament diameters and the desired diameter are less than 5 μm, which validates the reliability of the proposed method.

## 1. Introduction

In recent years, three-dimensional (3D) printing technology has been regarded as a promising method to fabricate in-vitro-functionalized tissues and organs, which provide possible alternative treatments for various diseases in the fields of regenerative medicine and tissue engineering [1,2]. It can manufacture various complex structures with different biomaterials via a predefined layer-by-layer printing process and shows excellent flexibility, efficiency, and repeatability [3,4].

Common methods for 3D printing are inkjet [5,6], extrusion [7,8], photopolymerization [9,10], etc. Currently, extrusion printing is the most frequently adopted method for 3D printing because it shows great compatibility for printing various materials for biofabrication with a wide range of viscosity, and the printing process is easy to operate [3,8]. The typical feature of extrusion 3D printing is that the materials are extruded from a cartridge through a channel or nozzle with a diameter in the range of several hundred micrometers under the action of air pressure or a plunger. Continuous filament with a diameter of several hundred micrometers can be extruded and then deposited onto the platform with a predefined trajectory, by which a complex 3D structure can be constructed. 

Many studies have been conducted based on extrusion 3D printing. Lee et al. [11] prepared scaffolds with PLCL and adipose-tissue-derived decellularized extracellular matrix hydrogel through extrusion 3D printing. Their research confirmed that the hydrogel–PLCL scaffolds could promote the differentiation of endothelial cells and their recruitment. Park et al. [12] fabricated a bone-like scaffold with melt blends of β-tricalcium phosphate (β-TCP) and polycaprolactone (PCL) by extrusion 3D printing. The cell differentiation of mouse mesenchymal stem cells was investigated with the scaffold. Khorsandi et al. [13] considered 3D printing technology as a promising technology in rapidly prototyping microfluid chips and sensors. He et al. [14] printed a porous PLCL scaffold by extrusion 3D printing. Great mechanical properties and biocompatibility were shown on the PLCL scaffolds, which were treated with alkali and coated with collagen type I. Merceron et al. [15] developed a complex tissue construct that comprised a mechanically heterogeneous polymeric scaffold by extrusion 3D printing. Thermoplastic polyurethane and PCL were utilized to provide mechanical stability for the printed structure. Kang et al. [16] fabricated cartilage, a mandible bone, and skeletal muscle by extrusion 3D printing. PCL and Pluronic F127 were printed along with cell-laden hydrogel to confine the shape of the printed scaffolds. The printed tissue constructs showed high vascularization and cell viability. Byambaa et al. [17] utilized extrusion 3D printing to develop vascular bone tissue constructs. Two gelatin methacryloyl-based hydrogels were synthesized and optimized to support the vasculogenesis and osteogenesis of the printed constructs. 

Although plenty of valuable research has been conducted based on extrusion 3D printing, research on the printing accuracy of extrusion 3D printing remains insufficient [14,18]. The printing accuracy of extrusion 3D printing is highly related to the diameter accuracy of the printed filament, which reflects the deviation between its actual diameter and the designed value. Table 1 compares the filament diameter deviations of recent research. It can be seen that diameter deviation was generally in the range of 15~25 μm. The overall accuracy of the printed structure can deteriorate significantly due to error accumulation during the layer-by-layer deposition process. Therefore, the deviation of filament diameter should be as small as possible.

The accuracy of the filament diameter is directly dependent on process parameters (extrusion force, temperature, etc.), the rheological properties of the materials, and the extrusion channel design of the print head [22,23]. Generally, the filament diameter is larger than the nozzle diameter due to the die swell phenomenon, and the filament diameter can be regulated by adjusting the process parameters. However, improper process parameters may fail to print continuous filament. Additionally, the rheological properties for a certain material varies with temperature variation. This means the temperature of the printing system should be well-controlled. This makes the accurate control of filament diameter extremely challenging. However, research on the relationship between filament diameter accuracy and process parameters of extrusion 3D printing has seldom been reported. In existing research, the trial-and-error method has been widely adopted when searching for proper process parameters. To acquire the desired filament diameter, the process parameters should be adjusted repeatedly with different parameter combinations, which is not only time-consuming but also lacks reliability and repeatability. Hence, the demand for a method to guide process parameter design is urgent to improve printing accuracy of the filament diameter.

This research proposes a process parameter design method based on experimentation to improve the accuracy of the printed filament diameter. A novel extrusion 3D printing device was developed to precisely control the process parameters of temperature and extrusion force, by which the influence of process parameters and the rheological properties on the printing accuracy of a certain material can be investigated efficiently. Moreover, a case study of PLCL was conducted to detail the design procedure of the proposed method. The relationship between process parameters and the accuracy of filament diameter was experimentally evaluated and theoretically discussed, which can guide future process parameter design for desired filament diameter. 

## 2. An Experiment-Based Process Parameter Design Method

**Figure 1:**
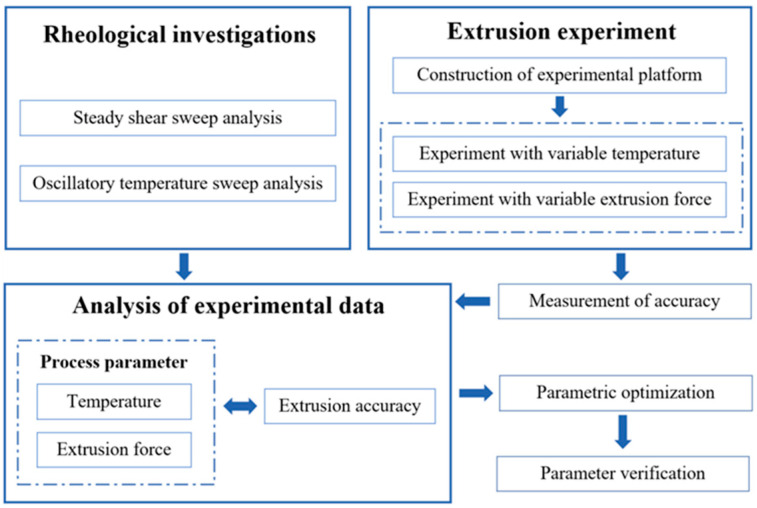
Schema of the experiment-based process parameter design method.

Figure 1 illustrates the schema of a process parameter design method based on experimentation. The proposed method consisted of three key procedures. Firstly, the rheological properties of the material were evaluated, such as the viscosity with varying temperatures and the complex modulus with varying shear rates. Then, to acquire the relationship between the process parameters and the diameter of the printed filament, temperature and the extrusion force were selected as two crucial parameters, and a novel experiment apparatus was developed by which these two process parameters were investigated to ensure the practicality and efficiency of the method. According to the experimental data, the influence rule of the process parameters on the diameter of the filament could be acquired, and the appropriate process parameters for the desired filament diameter could be determined efficiently. 

### 2.1. Rheological Investigation

Most of the materials adopted in extrusion 3D printing are pseudoplastic fluids that have complex rheological properties. The rheological parameters of materials, mainly including viscosity (*η*), storage modulus (*G′*), and loss modulus (*G″*), are the preconditions to theoretically analyze the extrusion 3D printing process [21]. The viscosity of pseudoplastic fluids always changes with varying temperature and shear rates. The fluid flow status of materials inside the printing nozzle can be easily affected by process parameters during extrusion 3D printing. Thus, the *η* curves of materials with varying temperatures and shear rates are indispensable to clarify the printing process with a certain material. Viscoelastic behavior is another unique characteristic of materials that means the materials have both viscosity and elasticity at the same time. This characteristic causes the diameter to increase during the extrusion printing process of polymers. To improve the accuracy of filament diameters, viscoelasticity must be taken into consideration. *G′* and *G″* are two common parameters that reflect viscoelasticity that are generally utilized to investigate the die swell phenomenon when filament is extruded from the print nozzle. Therefore, rheological investigations of the material should be first conducted before the design of process parameters for a certain material. 

**Figure 2:**
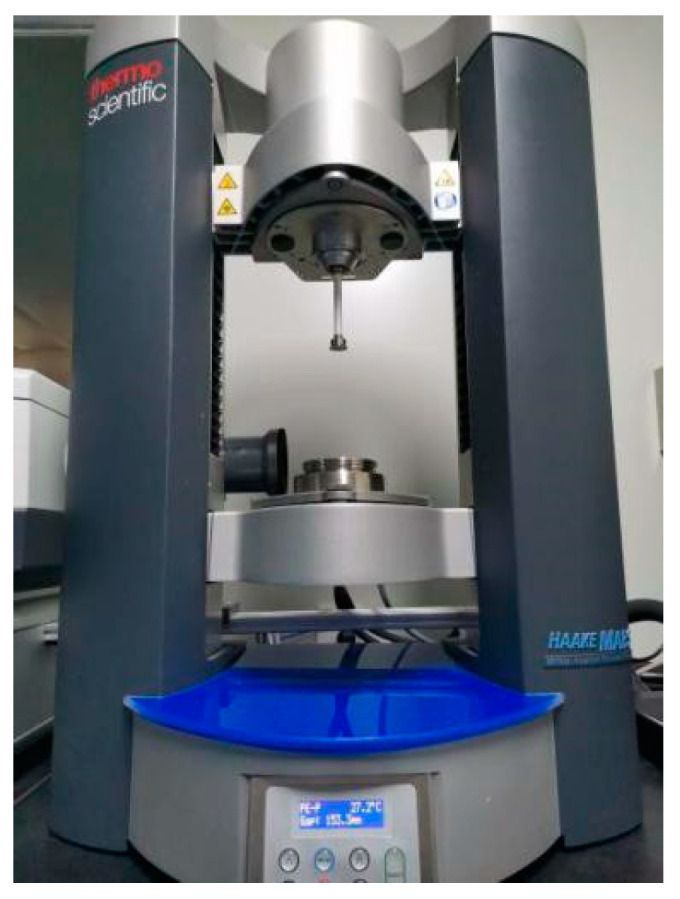
Rotation rheometer.

As shown in Figure 2, a rotation rheometer (HAAKE MARS III, Thermo Fisher Scientific, Shanghai, China) was employed in this study to investigate the rheological properties of the materials. Two analysis modes were utilized for this research: the steady shear sweep analysis mode and the oscillatory temperature sweep analysis mode. The steady shear sweep analysis was first performed at a constant temperature to obtain the *η* curves with varying shear rates. Then, the oscillatory temperature sweep analysis was conducted to evaluate the temperature-dependent *G′* and *G″*.

### 2.2. Extrusion Experiment

**Figure 3:**
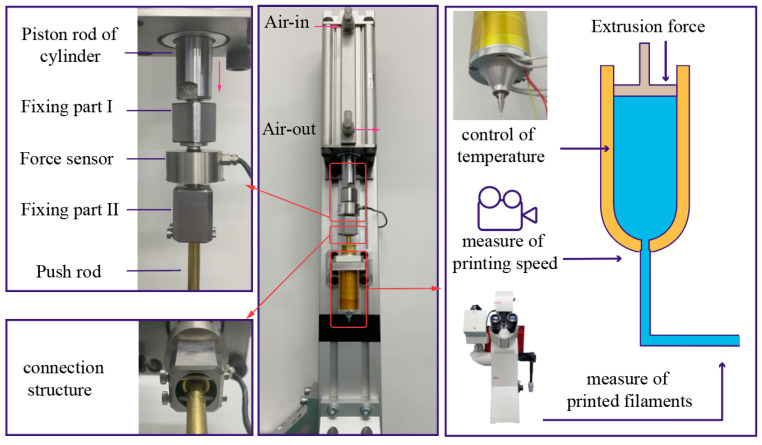
Process of the extrusion experiment.

The extrusion force acting on the material and the temperature of the material are two of the most significant process parameters of extrusion 3D printing. To investigate their influence on the accuracy of filament diameter, a novel extrusion 3D printing device that could precisely control extrusion force and temperature was designed. Existing extrusion 3D printers are usually driven by compressed air or a plunger actuated by a ball screw. The first drive type, due to the compressibility of air, is rather challenging for accurate control of the extrusion force. Additionally, for some kinds of materials that can only be printed at a high extrusion force, the pressurized-air-actuated print head cannot provide adequate extrusion force. Though the second drive type is capable of providing adequate extrusion force, it can only control the moving distance of the plunger, and the extrusion force cannot be controlled accurately. In order to overcome this limitation, a pneumatic-cylinder-actuated extrusion 3D printing device was designed, as shown in Figure 3. The extrusion force and temperature can be controlled precisely and separately with this extrusion 3D printing device.

Specifically, as illustrated in Figure 3, to precisely measure the extrusion force acted on the material, a force sensor is set between the piston rod of the pneumatic cylinder and the push rod of the print head. Two fixing parts are designed to connect the two rods and the force sensor. During the extrusion process, high-pressure air enters the inlet at the top of the pneumatic cylinder, while the air in the other end of the piston is pushed out of the pneumatic cylinder from the outlet. Then, the piston rod of the pneumatic cylinder moves down and presses on the force sensor, and the extrusion force can be displayed and recorded. Based on feedback from the force sensor, the extrusion force can be controlled by changing the air supply pressure.

To control the temperature of the materials during the printing process, a print head with a temperature control system was designed. The temperature control system consists of two heat bars and a Pt temperature sensor to record and adjust the temperature. After the material is heated to melting, the push rod is actuated to extrude the material, and the filament is extruded out of the nozzle.

To measure the speed of the filaments during the print process, a video camera recorder was used to record the experiment process. Then, the speed of printing was calculated by analyzing the records of the PLCL printing process frame by frame.

To measure the diameters of the printed filaments, the pictures of the printed filaments were first recorded using an optical microscope (Leica Microsystems DMI8, Wetzlar, Germany). Then, the sizes of the filaments in the pictures were calculated by comparing them with a scale bar. Three points for each filament were selected to be measured during the measurement process. Lastly, the diameters of each filament were calculated by averaging the three values.

### 2.3. Process Parameter Design

To obtain the proper process parameters for the extrusion 3D printing process of the desired diameters, the obtained data were further studied after the extrusion experiment, and the rheological investigations were finished. The rheological properties of material and hydromechanics were considered comprehensively to explain the experimental phenomenon. Then, the flow status of material was taken into consideration to explain the experimental results so that the inner mechanism of the variation trend of filament diameter with varying temperatures and extrusion forces could be clarified. Ultimately, the process parameters for the desired diameter of printing filament could be acquired efficiently through the investigation process mentioned above.

## 3. Case Study of Process Parameter Design for PLCL

PLCL is a kind of widely applied material in biofabrication with excellent biocompatibility and mechanical properties at room temperature. It is conducive to maintaining the biological activity of cells and providing tissue mechanical microenvironment support for cell survival. These characteristics make PLCL popular in the field of biological scaffold manufacturing. This paper used PLCL as a case study to detail the proposed method for process parameter design and optimization, and a set of appropriate process parameters for extrusion 3D printing was acquired, which proved the validity and feasibility of the proposed experiment-based process parameter design method.

### 3.1. Rheological Properties of PLCL

PLCL 50:50 pellets were purchased from Jinan Daigang Biomaterial Co., Ltd. (Jinan, China). For the rheological investigation and extrusion experiments, the PLCL pellets were firstly heated at 140 °C for more than 2 h until the pellets were melted and could be considered as ink for the extrusion 3D printing.

**Figure 4:**
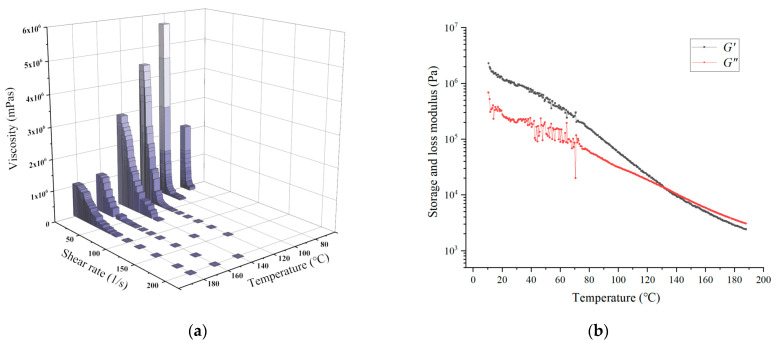
The rheological properties of PLCL: (**a**) viscosities of PLCL with varying temperatures and shear rates; (**b**) complex modulus curves of PLCL with varying temperatures.

To investigate the effects of varying shear rates and temperatures on the *η* of the PLCL melts, steady shear sweeps were performed from 1 to 200 1/s at several temperatures. These temperatures were selected from 80 °C to 180 °C with an increment of 20 °C. It can be seen from Figure 4a that the *η* dropped sharply with the increase of shear rate and then trended to steady with further growth of the shear rate. This phenomenon was derived from the disentanglement of polymer chains during shear flow. The *η* also decreased quickly when the temperature increased from 100 °C to 160 °C, while it only declined slightly when the temperature increased from 160 °C to 180 °C. This phenomenon was attributed to the molecules containing higher thermal energy to overcome the attractive forces binding them together when the temperature of the PLCL melt increased, which consequently decreased the viscosity. To obtain the *G′* and *G″* curves for varying temperatures, the PLCL melt was subjected to a temperature ramp in the range from 190 ℃ to 10 ℃ with a decrement rate of 0.1 °C/s. Figure 4b shows the *G′* and *G″* curves of PLCL with varying temperatures at the shear rate of 1 1/s. It can be seen from Figure 4b that the *G′* was higher than the *G″* at room temperature, which indicates that elasticity played a dominant role in this condition. With the increase in temperature, both the *G′* and *G″* declined, and the *G′* declined faster. When the temperature exceeded 130 °C, the *G″* was higher than the *G′*, which means viscosity played a dominant role. Additionally, when the temperature was lower than 80 °C, the *G′* and *G”* curves fluctuated wildly. It indicated that PLCL melts are difficult to flow when the temperature is below 80 °C.

### 3.2. Influence of Process Parameters on Printing Accuracy

In this paper, a nozzle diameter of 100 μm was adopted for the print head. To investigate the relationship between filament diameter and two process parameters, the diameters of printed filaments with varying temperatures were measured while the extrusion force was set to three constant values: 20, 40, and 60 kg. The printing speed of each printing process was also recorded during the experiment. Each extrusion process was conducted three times.

**Figure 5:**
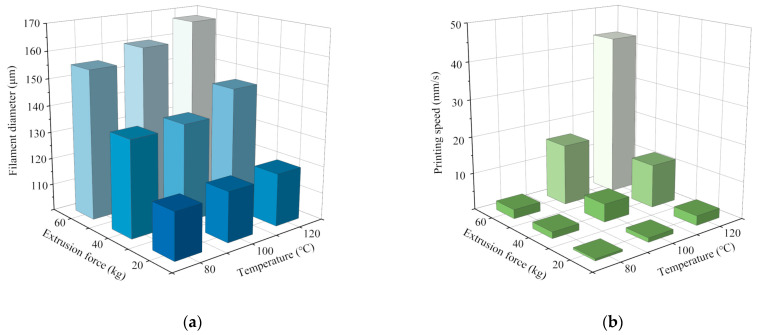
Influence of temperature on the extrusion process: (**a**) filament diameters of PLCL with varying extrusion forces and temperatures; (**b**) print speed of filaments with varying extrusion forces and temperatures.

**Figure 6:**
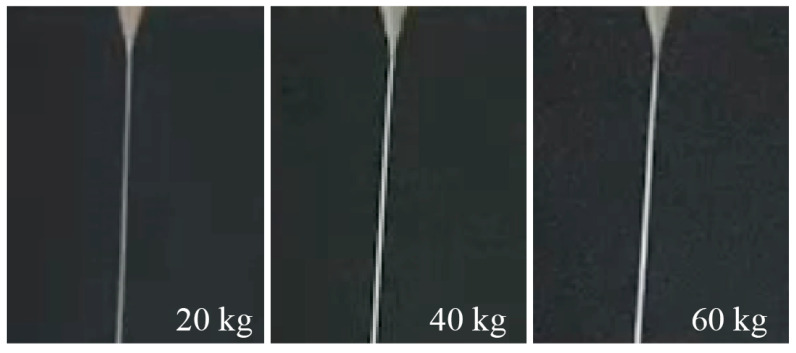
Images of the printing filaments at 100 °C.

Figure 5 presents the average values of filament diameters and printing speeds in each printing process. It can be seen that the filament diameters were all larger than the nozzle diameter of 100 μm due to the die swell phenomenon, and the filament diameters varied with the variation of extrusion force and temperature. The maximum filament diameter reached 167 μm, while the minimum filament diameter was 117 μm with the temperature range of 80~120 °C and the extrusion force range of 20~60 kg. It indicated that the filament diameter could be controlled by changing the process parameters. Figure 6 presents several pictures of printing filaments.

To explain the variation of filament diameters in Figure 5a, the printing speed must be taken into consideration. According to the theory of polymer conformation, during the extrusion 3D process of PLCL melts, the die swell phenomenon always occurs and is related to the entropic elasticity [24]. Conformational entropy is a kind of entropy which is related to the number of conformations of a molecule. Before the PLCL melts flow into the pipeline of the nozzle, the polymer conformation of each PLCL molecule is a random coil. The number of conformations of each random coil is always at maximum value, which means the PLCL melts possess maximum conformational entropy before entering the pipeline. When the melt enters into the pipeline, the velocity gradient of the PLCL flow in the pipeline changes the orientation of molecular chains and, consequently, leads to a decrease in the number of conformations of the molecules. Thus, the conformational entropy of the PLCL melts also decreases. Meanwhile, the physical entanglements resulting from the orientation of molecular chains in the pipeline partly relax (disentanglement) due to the Brownian motion of the molecules, which means that the decline of entropy caused by the velocity gradient partly recovers. The longer it takes in the pipeline, the more entropy it recovers due to the disentanglement of PLCL polymer chains. When the PLCL melts leave the pipeline, the remaining physical entanglements relax, and the orientation of the molecules no longer exists. The polymer conformation returns to the random coil, and the number of conformations of molecules returns to the maximum value. The increase in conformational entropy leads to an increase in filament diameter, which is called the die swell phenomenon. However, the entropy of the PLCL melts is recovered inside the pipeline because of Brownian motion. The shorter the time in the nozzle, the greater the amount of remaining physical entanglement and the stronger the die swell phenomenon of the PLCL. As shown in Figure 5b, the increase of printing speed caused by increasing extrusion force and temperature led to a decline in the time spent in the pipeline. The relaxation of the orientation of molecular chains caused by Brownian motion decreased, which led to a larger remaining entropy of the PLCL extruded from the nozzle. The diameters of printed filaments were much larger than the nozzle in this condition. This explains the reason why the diameter of printed filaments declined with the decrease in extrusion force and temperature.

To further explain the variation of printing speeds in Figure 5b, the flow of the PLCL melts should be taken into consideration. The melts in the nozzle can be regarded as an incompressible fluid with constant density. The Navier–Stokes equation of incompressible viscous fluid in a cylindrical coordinate system can be used to discuss the flow of the PLCL melts in the nozzle [25]:

(1) $$\frac{dw_{r}}{dt}-\frac{w_{\theta }^{2}}{r}=-\frac{1}{\rho }\frac{\partial p}{\partial r}+f_{r}+\nu \left({▽}^{2}w_{r}-\frac{w_{r}}{r^{2}}-\frac{2}{r^{2}}\frac{\partial w_{\theta }}{\partial \theta }\right)\frac{dw_{r}}{dt}-\frac{w_{\theta }^{2}}{r}\;=-\frac{1}{\rho }\frac{\partial p}{\partial r}+f_{r}+\nu \left({▽}^{2}w_{r}-\frac{w_{r}}{r^{2}}-\frac{2}{r^{2}}\frac{\partial w_{\theta }}{\partial \theta }\right);\;$$

(2) $$\frac{dw_{\theta }}{dt}-\frac{w_{r}w_{\theta }}{r}=-\frac{1}{\rho r}\frac{\partial p}{\partial \theta }+f_{\theta }+\nu \left({▽}^{2}w_{\theta }+\frac{2}{r^{2}}\frac{\partial w_{r}}{\partial \theta }-\frac{w_{\theta }}{r^{2}}\right);$$

(3) $$\frac{dw_{z}}{dt}=-\frac{1}{\rho }\frac{\partial p}{\partial z}+f_{z}+\nu {▽}^{2}w_{z},$$

The Reynolds number of the flow in the nozzle is lower than 2300. This means the flow in the nozzle can be considered a laminar flow. For laminar flow in the pipeline, $w_{\theta }=0$ and $w_{r}=0$. The only existing velocity in the Navier–Stokes equation is $\;w_{z}=w\left(r,\;\theta \right)$. Due to the small longitudinal dimension of the pipeline, the volume force can also be ignored. The flow of the PLCL melt in the nozzle during the extrusion process can be considered as an incompressible viscous fluid driven by a pressure gradient. The means the flow velocity is $w_{m},$ and the pressure gradient is expressed as followed [22]:

(4) $$w_{m}=-\frac{dp}{dz}\frac{r_{0}{}^{2}}{8\eta }.$$

The expression $\frac{dp}{dz}$ represents the pressure gradient of the flow in the nozzle. Equation (4) indicates that either an increase in pressure gradient or a decrease in viscosity leads to an increase in the mean flow velocity of the melt. During the extrusion process, a higher extrusion force acting on the PLCL melts led to a stronger pressure gradient. According to Figure 4a, the viscosity of PLCL decreased with the increase in temperature. Therefore, the speed of the filament increased with the growth in temperature and extrusion force.

**Figure 7:**
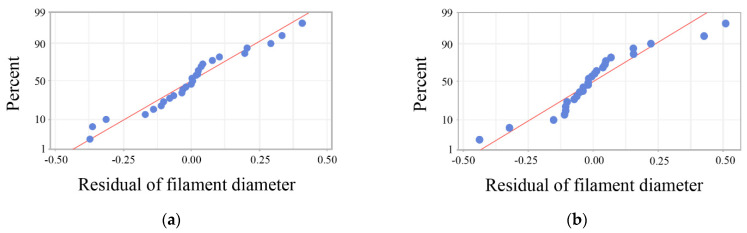
Residual plots of filament diameters: (**a**) normal probability plot of filament diameters; (**b**) normal probability plot of printing speed.

To verify the robustness of the experimental data, an analysis of variance for filament diameters and printing speeds was conducted with Minitab. Normal probability plots of filament diameter and printing speed were first obtained in Figure 7. In the normal probability plots, the closer the points lay to a straight line, the better the data fit the normal distribution. It can be seen from Figure 7 that both the residual data of filament diameter and printing speed were approximately normally distributed, which verifies the validity of the experimental data. Then, an F-test was conducted to test for statistical significance at a significance level (α) of 0.05. Thus, the influence of sampling error on the observed effect could be measured. Critical data of the variance analysis are shown in Table 2. By comparing the observed value of F (F-value) with the critical value of F (F_0.05_) determined from tables, it is indicated that the F-values of both temperature and extrusion force were higher than the F_0.05_. Hence, the influences of these two parameters on filament diameters and printing speeds reached a significant level, which means the observed effects of the data were not caused by the sampling error at a 95% confidence interval.

### 3.3. Design of Process Parameters of PLCL Extrusion 3D Printing

The design procedure of the process parameters can be divided into two parts: finding the printable range of process parameters and determining a set of proper process parameters for printing filaments with the desired diameter.

To obtain the printable range of extrusion force and temperature for PLCL, the rheological properties and the experimental phenomena should be taken into consideration comprehensively. According to Figure 5b, the printing speed of PLCL filaments decreased with the decrease in temperature and extrusion force. When the temperature was lower than 80 °C, the PLCL could not be extruded from the nozzle because of its high viscosity and low fluidity at such a low temperature, as shown in Figure 4. Additionally, the extrusion of PLCL melt was also unsuccessful when the extrusion force was below 20 kg. According to Equation (4), a lower extrusion force caused a lower pressure gradient, which consequently resulted in the PLCL melt becoming difficult to print. The printing temperature of PLCL with extrusion 3D printing should be higher than 80 °C, and the extrusion force should exceed 20 kg. 

**Figure 8:**
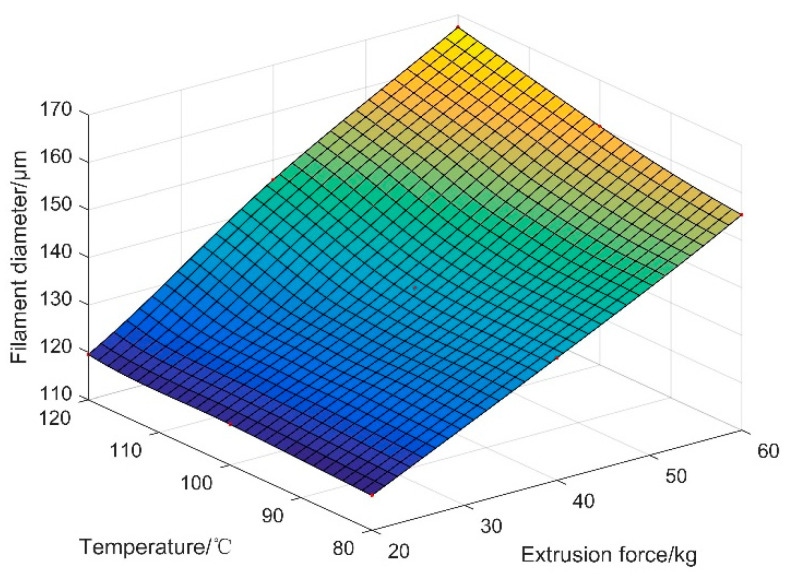
Surface fitting of the experimental data.

To control filament diameter and guide process parameter design in the printable range, the relationship between the diameters of printed filaments and process parameters is further investigated in this section. According to Figure 5a, the diameters of printed filaments in each printing experiment had a clear correspondence with the temperature and extrusion force. To discover the printing parameters for a filament with a certain diameter, the experimental data in Figure 5a were fitted to a surface, as shown in Figure 8. The diameter of the filament to print can be predicted, and the proper process parameters for filaments with a certain size can be determined through the fitting surface.

### 3.4. Experimental Verification

In order to verify the effectiveness and reliability of the proposed process parameter design method, a group of print trials was conducted to fabricate PLCL filaments of the desired sizes. Five filament diameters of 120, 130, 140, 150, and 160 μm were selected as the target diameters of the printing experiments.

To fabricate PLCL filaments of these five diameters, the corresponding extrusion force and temperature were first selected from the fitting surface in Figure 8. The process parameters for certain filament diameters are shown in Table 3. During the extrusion experiment, the PLCL pellets were first put into the print head and heated to 91 °C for 2 h. When the PLCL completely became melts, the air pressure entering the cylinder was then controlled until the extrusion force recorded by the force sensor reached 22.2 kg. Meanwhile, the piston rod of the cylinder was controlled to push the material inside the print head, and the PLCL filament was pushed out of the nozzle. Moreover, the printed filaments were collected and recorded by an optical microscope. The detailed printing procedure of the other four diameters was identical with that of the 120 μm filament.

**Figure 9:**
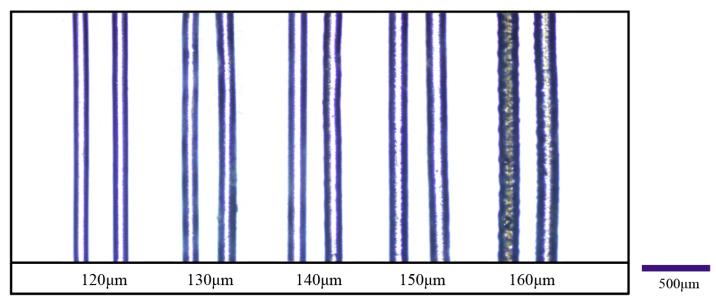
Printed filaments of PLCL with designed process parameters.

Figure 9 shows optical microscope pictures of the filaments printed in the experiment. The diameters of the printed filaments were measured by comparing filaments in the picture with a scale bar, and the deviation between the desired diameter and the actual diameter of printed filaments was evaluated. It was shown that all the deviations were less than 5 μm for each designed diameter. The minimum deviation of filament diameter of the references shown in Table 1 was larger than 15 μm, which is much higher than that of our research. Additionally, the five different diameters of filament can be printed with a 100 μm printing nozzle by adjusting the process parameters. This verified the feasibility of the proposed method to improve the accuracy of extrusion 3D printing.

## 4. Conclusions

This paper presented an experiment-based process parameter design method that aimed to control the filament diameter of extrusion 3D printing. PLCL was used as a case study to detail the design procedure. The main conclusions in this paper are as follows:

(1) To quickly determine the proper process parameters for the desired filament diameters, an experiment-based process parameter design method was proposed to facilitate the process parameter design for extrusion 3D printing;
(2) A novel extrusion 3D printing device was designed to precisely control extrusion force and temperature, by which the relationship between the process parameters and the filament diameter could be obtained efficiently and conveniently;
(3) The process parameter design for PLCL was conducted as a case study of the proposed method. The printable range of the process parameters for PLCL was acquired, and a fitting surface of the experimental data was calculated to facilitate process parameter design.
(4) A group of print trials was conducted, and the deviations between the actual diameters of the printed filaments and the desired diameters were less than 5 μm, which verified the feasibility and reliability of the proposed method.

## Figures and Tables

**Table 1:** Results of printed PLCL filaments in recent research.

Reference	Printing Method	Material	Minimum Diameter and Deviation (μm)
[14]	Extrusion 3D printing	PLCL	244.80 ± 16.71
[19]			225.24 ± 15.27
[20]			383.81 ± 23.13
[21]			343.79 ± 21.21

**Table 2:** Analysis of variance for filament diameters and printing speed.

Factor	Source	F_0.05_	F-Value
Filament diameter	Temperature (°C)	3.56	3065.68
	Extrusion force (kg)		78,988.19
	2-way interactions	2.93	483.10
Printing speed	Temperature (°C)	3.56	14,103.97
	Extrusion force (kg)		17,839.08
	2-way interactions	2.93	6303.12

**Table 3:** Process parameters of extrusion 3D printing.

Diameter (μm)	Temperature (°C)	Extrusion Force (kg)
120	91.0	22.2
130	106.4	31.2
140	95.4	43.8
150	113.2	46.7
160	116.2	54.4

## Data Availability

Not applicable.
